# Poly[tetra­aqua-μ_4_-bromido-di-μ_2_-bromido-μ_2_-hydroxido-di-μ_3_-iso­nicotinato-tetra-μ_2_-isonicotinato-tetra­copper(I)dithulium(III)]

**DOI:** 10.1107/S1600536808028675

**Published:** 2008-09-13

**Authors:** Guo-Ming Wang, Zeng-Xin Li, Qing-Hua Zheng, Hui-Luan Liu

**Affiliations:** a Department of Chemistry, Teachers College of Qingdao University, Qingdao, Shandong 266071, People’s Republic of China

## Abstract

A new thulium(III)–copper(I) heterometallic coordination polymer, [Cu_4_Tm_2_Br_3_(C_6_H_4_NO_2_)_6_(OH)(H_2_O)_4_]_*n*_, has been prepared by a hydro­thermal method. The Tm and both Cu atoms lie on mirror planes. The Tm atom is seven-coordinate with a capped distorted trigonal–prismatic coordination geometry, while the Cu atoms adopt trigonal CuBrN_2_ and tetra­hedral CuBr_3_N coordination modes, respectively. The Cu atom in the trigonal coordination environment is disordered over two sites of equal occupancy. The crystal structure is constructed from two distinct units of dimeric [Tm_2_(μ_2_-OH(IN)_6_(H_2_O)_4_] cores (IN = isonicotinate) and one-dimensional inorganic [Cu_4_Br_3_]_*n*_ chains, which are linked together, forming heterometallic Cu–halide–lanthanide–organic layers.

## Related literature

For background to the structures and applications of heterometallic lanthanide–transition metal polymers, see: Benelli & Gatteschi (2002 ▶); Shibasaki & Yoshikawa (2002 ▶); Zhao *et al.* (2004*a* ▶,*b* ▶); Guillou *et al.* (2006 ▶); Wang *et al.* (2006 ▶). For some examples of heterometallic lanthanide–transition metal extended architectures, see: Ren *et al.* (2003 ▶); Prasad *et al.* (2007 ▶); Cheng *et al.* (2008 ▶).
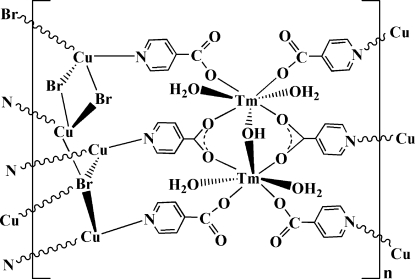


## Experimental

### 

#### Crystal data

[Cu_4_Tm_2_Br_3_(C_6_H_4_NO_2_)_6_(OH)(H_2_O)_4_]
*M* *_r_* = 1653.43
Orthorhombic, 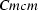

*a* = 19.1815 (2) Å
*b* = 6.6973 (4) Å
*c* = 34.7044 (5) Å
*V* = 4458.3 (3) Å^3^
*Z* = 4
Mo *K*α radiation
μ = 8.58 mm^−1^
*T* = 295 (2) K
0.16 × 0.09 × 0.08 mm

#### Data collection

Bruker APEXII area-detector diffractometer
Absorption correction: multi-scan (*SADABS*; Sheldrick, 1996 ▶) *T*_min_ = 0.341, *T*_max_ = 0.547 (expected range = 0.313–0.503)
17231 measured reflections
2408 independent reflections
2090 reflections with *I* > 2σ(*I*)
*R*_int_ = 0.037

#### Refinement

*R*[*F* ^2^ > 2σ(*F* ^2^)] = 0.027
*wR*(*F* ^2^) = 0.064
*S* = 1.11
2408 reflections
179 parameters
6 restraints
H-atom parameters constrained
Δρ_max_ = 0.83 e Å^−3^
Δρ_min_ = −0.87 e Å^−3^

### 

Data collection: *APEX2* (Bruker, 2002 ▶); cell refinement: *SAINT* (Bruker, 2002 ▶); data reduction: *SAINT*; program(s) used to solve structure: *SHELXS97* (Sheldrick, 2008 ▶); program(s) used to refine structure: *SHELXL97* (Sheldrick, 2008 ▶); molecular graphics: *SHELXTL* (Sheldrick, 2008 ▶); software used to prepare material for publication: *SHELXTL*.

## Supplementary Material

Crystal structure: contains datablocks global, I. DOI: 10.1107/S1600536808028675/sj2536sup1.cif

Structure factors: contains datablocks I. DOI: 10.1107/S1600536808028675/sj2536Isup2.hkl

Additional supplementary materials:  crystallographic information; 3D view; checkCIF report

## Figures and Tables

**Fig. 1.**
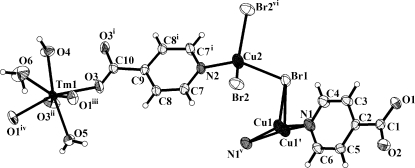
The molecular structure of 1, showing the atom-numbering scheme. Displacement ellipsoids are drawn at the 50% probability level. [Symmetry codes: (i) -x, y, z; (ii) x, y, 1/2 - z; (iii) x, 1 - y, 1 - z; (iv) x, 1 - y, -1/2 + z; (v) x, 2 - y, 1 - z; (vi) -x, 1 - y, 1 - z].

**Fig. 2.**
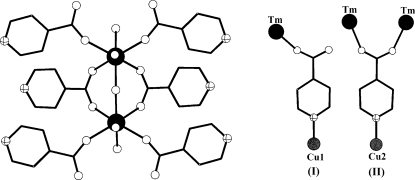
Dimeric [Tm2(µ2-OH)(IN)6] fragment and the coordination modes of IN- found in 1.

**Fig. 3.**
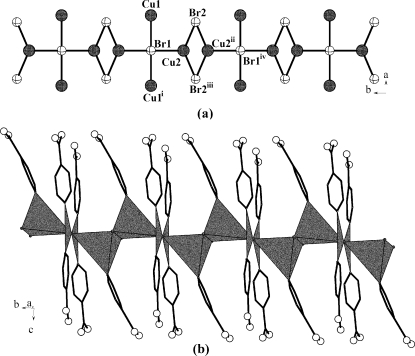
One-dimensional infinite [Cu4Br3]n chains along b axis (a), and polyhedral view of the [Cu4Br3N6]n chains. [Symmetry codes: (i) -x, 2 - y, 1 - z; (ii) x, 1 - y, 1 - z; (iii) -x, 1 - y, 1 - z; (iv) x, -1 + y, z].

**Fig. 4.**
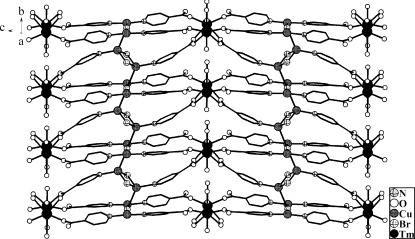
View of the two-dimensional layer structure of 1.

## supplementary crystallographic information

### Comment

The rational design and synthesis of heterometallic lanthanide(Ln)–transition metal(TM) compounds have attracted increasing attention in recent years, not only because of their intriguing variety of architectures and topologies but also owing to their potential applications in luminescence, magnetism, bimetallic catalysis, and molecular adsorption. (Benelli & Gatteschi, 2002; Shibasaki & Yoshikawa, 2002; Zhao *et al.*, 2004*a*,*b*; Guillou *et al.*, 2006; Wang *et al.*, 2006). So far, most work has been focused on the assembly of homometallic Ln and TM compounds, while the construction of heterometallic Ln–TM extented architectures is still a challenge (Ren *et al.*, 2003; Prasad *et al.*, 2007; Cheng *et al.*, 2008). This may be attributed to the different competitive reactions of Ln and TM metals with the same ligand, which often results in the formation of the homometallic compounds rather than heterometallic ones. Generally, the Ln ions prefer O-donors, while TM ions have stronger tendency to coordinate to N-donors. Therefore, isonicotinic acid (HIN) has been chosen here as the multifunctional bridging ligand to construct new hetero-Ln–TM complexes. The title compound [Tm_2_Cu_4_Br_3_(µ2-OH)(C_6_H_4_NO_2_)_6_(H_2_O)_4_]_n_ (1) is reported here and displays novel two-dimensional coordination features.

In the asymmetric unit, the Tm1 atom, occupying a special position on a mirror plane (Fig. 1), is seven-coordinated and has a capped trigonal–prismatic coordination environment comprising two coordinated water molecules, one µ_2_-OH and four carboxylate oxygen atoms from four IN^-^ ligands. The Tm—O bond lengths range from 2.193 (2) to 2.449 (5) Å. Both Cu1 and Cu2 atoms occupy special positions on two crystallographic mirror planes, one perpendicular (*z* = 0) and the other parallel to the *c* axis. The Cu1 atom is three-coordinate with one µ_4_-Br1 and two N atoms from two bridging IN^-^ moieties, while the Cu2 center is coordinated to one µ_4_-Br1, two µ_2_-Br2 atoms and one N atom from one IN^-^ ligand that defines a distorted tetrahedral geometry. The Cu—N and Cu—Br distances are in the range 1.928 (3)–2.020 (4) Å and 2.518 (9)–2.688 (7) Å, respectively. Although copper(II) salts were used as starting materials, the Cu centers in the product are in the +1 oxidation state. This is attributed to a reduction reaction occurring under the hydrothermal conditions used.

The framework of 1 is constructed from two subunits, dimeric [Tm_2_(µ_2_-OH)(IN^-^)_6_] (Tm_2_) cores and inorganic [Cu_4_Br_3_]_n_ chains. As shown in Fig. 2, two crystallographically identical Tm(III) ions are linked by one bridging hydroxo group and six IN^-^ ligands to form the dimeric Tm_2_ fragment, in which the IN^-^ ligand adopts two different coordination modes. The Tm···Tm distance is 4.071 (1) Å. The Cu(I) centers, however, are bridged by µ_4_-Br and µ_2_-Br atoms to form one-dimensional inorganic [Cu_4_Br_3_]_n_ chains (Fig. 3). Interestingly, such one-dimensional copper halide chains appear to be constructed directly from corner- and edge-sharing tetrahedral Cu(2)Br_3_N units, decorated by the trigonal Cu(1)BrN_2_ groups protruding outside the chain. In addition, the Cu···Cu distance within the copper(I) halide chains is 2.688 (7) Å, less than twice the van der Waals radius of the Cu(I) ion (1.4) Å), indicating a strong Cu···Cu interaction. The linkages between dimeric Tm_2_ and inorganic [Cu_4_Br_3_]_n_ motifs through N—Cu bonds give rise to a novel two-dimensional hetero-Tm—Cu framework (Fig. 4). Adjacent sheets are further packed to form a three-dimensional supramolecular framework through O—H···O hydrogen bonds.

### Experimental

The title compound was synthesized under mild hydrothermal conditions. Typically, a mixture of Tm_2_O_3_ (0.5 mmol, 0.193 g), CuBr_2_ (0.089 g, 0.40 mmol), HIN (2.00 mmol, 0.247 g) and H_2_O (8 ml) was sealed in a 25 ml Teflon-lined steel autoclave and heated under autogenous pressure at 443 K for 8 days. The yellow block-like crystals obtained were recovered by filtration, washed with distilled water and dried in air.

### Refinement

H atoms bound to C atoms were placed in calculated positions, with C—H distances of 0.93 Å. All other H atoms were located in a difference Fourier map and treated as riding, with fixed *U*_iso_(H) = 1.2*U*_eq_(O). The H4 and H6C atoms lie close to a mirror plane, and were treated as disordered with constrained site occupancy factors of 0.25 and 0.5, respectively. Atom Cu1 was refined as disordered over two positions, each with 50% site occupancy.

### Crystal data

**Table:**

[Cu_4_Tm_2_Br_3_(C_6_H_4_NO_2_)_6_(OH)(H_2_O)_4_]	*F*(000) = 3144
*M**_r_* = 1653.43	*D*_x_ = 2.463 Mg m^−^^3^
Orthorhombic, *C**m**c**m*	Mo *K*α radiation, λ = 0.71073 Å
Hall symbol: -C 2c 2	Cell parameters from 6070 reflections
*a* = 19.1815 (2) Å	θ = 2.1–26.5°
*b* = 6.6973 (4) Å	µ = 8.58 mm^−^^1^
*c* = 34.7044 (5) Å	*T* = 295 K
*V* = 4458.3 (3) Å^3^	Block, yellow
*Z* = 4	0.16 × 0.09 × 0.08 mm

### Data collection

**Table:**

Bruker APEXII area-detector diffractometer	2408 independent reflections
Radiation source: fine-focus sealed tube	2090 reflections with *I* > 2σ(*I*)
graphite	*R*_int_ = 0.037
φ and ω scans	θ_max_ = 26.5°, θ_min_ = 2.1°
Absorption correction: multi-scan (SADABS; Sheldrick, 1996)	*h* = −24→24
*T*_min_ = 0.341, *T*_max_ = 0.547	*k* = −8→8
17231 measured reflections	*l* = −43→42

### Refinement

**Table:**

Refinement on *F*^2^	Secondary atom site location: difference Fourier map
Least-squares matrix: full	Hydrogen site location: inferred from neighbouring sites
*R*[*F*^2^ > 2σ(*F*^2^)] = 0.027	H-atom parameters constrained
*wR*(*F*^2^) = 0.064	*w* = 1/[σ^2^(*F*_o_^2^) + (0.0288*P*)^2^ + 11.1134*P*] where *P* = (*F*_o_^2^ + 2*F*_c_^2^)/3
*S* = 1.11	(Δ/σ)_max_ = 0.001
2408 reflections	Δρ_max_ = 0.83 e Å^−^^3^
179 parameters	Δρ_min_ = −0.87 e Å^−^^3^
6 restraints	Extinction correction: SHELXL97 (Sheldrick, 2008), Fc^*^=kFc[1+0.001xFc^2^λ^3^/sin(2θ)]^-1/4^
Primary atom site location: structure-invariant direct methods	Extinction coefficient: 0.00119 (4)

### Special details

**Table:**

Geometry. All e.s.d.'s (except the e.s.d. in the dihedral angle between two l.s. planes) are estimated using the full covariance matrix. The cell e.s.d.'s are taken into account individually in the estimation of e.s.d.'s in distances, angles and torsion angles; correlations between e.s.d.'s in cell parameters are only used when they are defined by crystal symmetry. An approximate (isotropic) treatment of cell e.s.d.'s is used for estimating e.s.d.'s involving l.s. planes.
Refinement. Refinement of *F*^2^ against ALL reflections. The weighted *R*-factor *wR* and goodness of fit *S* are based on *F*^2^, conventional *R*-factors *R* are based on *F*, with *F* set to zero for negative *F*^2^. The threshold expression of *F*^2^ > σ(*F*^2^) is used only for calculating *R*-factors(gt) *etc*. and is not relevant to the choice of reflections for refinement. *R*-factors based on *F*^2^ are statistically about twice as large as those based on *F*, and *R*- factors based on ALL data will be even larger.

### Fractional atomic coordinates and isotropic or equivalent isotropic displacement parameters (Å^2^)

**Table:**

	*x*	*y*	*z*	*U*_iso_*/*U*_eq_	Occ. (<1)
Tm1	0.106107 (10)	−0.00154 (3)	0.2500	0.01935 (10)	
Cu1	0.1384 (6)	1.0000	0.5000	0.037 (3)	0.50
Cu1'	0.1361 (6)	1.064 (2)	0.4997 (8)	0.038 (2)	0.25
Cu2	0.0000	0.63637 (13)	0.47157 (2)	0.0465 (2)	
Br1	0.0000	1.0000	0.5000	0.0344 (2)	
Br2	0.11106 (3)	0.5000	0.5000	0.04244 (17)	
O1	0.16098 (14)	1.0892 (4)	0.69667 (7)	0.0352 (6)	
O2	0.27354 (15)	1.1077 (5)	0.68216 (7)	0.0475 (8)	
O3	0.05791 (13)	0.2265 (5)	0.29248 (8)	0.0390 (7)	
O4	0.0000	−0.1250 (8)	0.2500	0.0389 (13)	
H4	0.0000	−0.2788	0.2600	0.047*	0.50
O5	0.19308 (19)	0.2314 (6)	0.2500	0.0365 (9)	
H5	0.2041	0.2871	0.2699	0.044*	
O6	0.1159 (2)	−0.3688 (7)	0.2500	0.0647 (15)	
H6B	0.1529	−0.2988	0.2500	0.078*	
H6C	0.1278	−0.4859	0.2380	0.078*	0.50
C1	0.2117 (2)	1.0891 (6)	0.67359 (9)	0.0278 (8)	
C2	0.19342 (19)	1.0649 (5)	0.63145 (9)	0.0244 (7)	
C3	0.1278 (2)	1.0044 (6)	0.62013 (11)	0.0307 (8)	
H3A	0.0932	0.9823	0.6384	0.037*	
C4	0.1139 (2)	0.9771 (6)	0.58161 (12)	0.0344 (10)	
H4A	0.0702	0.9295	0.5744	0.041*	
C5	0.2424 (2)	1.0999 (6)	0.60318 (10)	0.0303 (8)	
H5A	0.2873	1.1397	0.6098	0.036*	
C6	0.2246 (2)	1.0755 (6)	0.56502 (10)	0.0323 (8)	
H6A	0.2580	1.1008	0.5462	0.039*	
C7	0.0597 (2)	0.5415 (6)	0.39568 (11)	0.0307 (9)	
H7A	0.1015	0.5754	0.4076	0.037*	
C8	0.06218 (19)	0.4538 (5)	0.35956 (11)	0.0260 (8)	
H8A	0.1047	0.4302	0.3475	0.031*	
C9	0.0000	0.4016 (7)	0.34159 (13)	0.0216 (10)	
C10	0.0000	0.2775 (8)	0.30518 (13)	0.0240 (10)	
N1	0.16080 (18)	1.0165 (5)	0.55395 (9)	0.0340 (8)	
N2	0.0000	0.5802 (7)	0.41440 (12)	0.0275 (9)	

### Atomic displacement parameters (Å^2^)

**Table:**

	*U*^11^	*U*^22^	*U*^33^	*U*^12^	*U*^13^	*U*^23^
Tm1	0.01730 (14)	0.02826 (15)	0.01249 (13)	0.00208 (9)	0.000	0.000
Cu1	0.0466 (19)	0.052 (8)	0.0131 (15)	0.000	0.000	0.000 (8)
Cu1'	0.047 (2)	0.051 (7)	0.0145 (17)	−0.004 (4)	0.0008 (16)	0.001 (5)
Cu2	0.0465 (4)	0.0686 (6)	0.0243 (4)	0.000	0.000	−0.0111 (4)
Br1	0.0271 (4)	0.0500 (5)	0.0260 (4)	0.000	0.000	−0.0129 (3)
Br2	0.0286 (3)	0.0619 (4)	0.0368 (4)	0.000	0.000	0.0074 (3)
O1	0.0438 (16)	0.0448 (16)	0.0171 (13)	−0.0065 (14)	0.0069 (11)	0.0009 (12)
O2	0.0406 (17)	0.081 (2)	0.0212 (14)	−0.0224 (17)	−0.0013 (12)	−0.0077 (15)
O3	0.0250 (13)	0.0570 (18)	0.0348 (15)	0.0037 (13)	0.0023 (11)	−0.0254 (13)
O4	0.023 (3)	0.033 (3)	0.061 (4)	0.000	0.000	0.000
O5	0.046 (2)	0.051 (2)	0.0127 (17)	−0.021 (2)	0.000	0.000
O6	0.053 (3)	0.027 (2)	0.113 (5)	0.008 (2)	0.000	0.000
C1	0.039 (2)	0.031 (2)	0.0138 (16)	−0.0086 (17)	0.0020 (15)	0.0005 (15)
C2	0.034 (2)	0.0235 (16)	0.0161 (16)	−0.0035 (15)	−0.0005 (14)	−0.0006 (14)
C3	0.0290 (18)	0.042 (2)	0.0207 (18)	−0.0027 (17)	0.0035 (15)	0.0043 (15)
C4	0.030 (2)	0.050 (3)	0.024 (2)	−0.0011 (18)	−0.0018 (16)	−0.0005 (17)
C5	0.0307 (19)	0.039 (2)	0.0211 (18)	−0.0088 (17)	0.0013 (15)	−0.0008 (17)
C6	0.033 (2)	0.045 (2)	0.0188 (18)	−0.0045 (18)	0.0048 (15)	−0.0001 (17)
C7	0.028 (2)	0.040 (2)	0.024 (2)	−0.0044 (16)	−0.0016 (15)	−0.0091 (16)
C8	0.0226 (18)	0.032 (2)	0.0229 (18)	−0.0003 (14)	0.0006 (14)	−0.0065 (15)
C9	0.029 (2)	0.018 (2)	0.018 (2)	0.000	0.000	0.0000 (19)
C10	0.024 (2)	0.028 (3)	0.020 (2)	0.000	0.000	−0.003 (2)
N1	0.0339 (18)	0.051 (2)	0.0176 (15)	0.0022 (15)	0.0003 (13)	0.0007 (14)
N2	0.034 (2)	0.030 (2)	0.018 (2)	0.000	0.000	−0.0048 (19)

### Geometric parameters (Å, °)

**Table:**

Tm1—O4	2.197 (2)	O3—C10	1.243 (3)
Tm1—O1^i^	2.208 (2)	O4—Tm1^ix^	2.197 (2)
Tm1—O1^ii^	2.208 (2)	O4—H4	1.0867
Tm1—O5	2.284 (4)	O5—H5	0.8127
Tm1—O3^iii^	2.315 (2)	O6—H6B	0.8504
Tm1—O3	2.315 (2)	O6—H6C	0.9163
Tm1—O6	2.467 (5)	C1—C2	1.512 (5)
Tm1—H6B	2.1833	C2—C5	1.379 (5)
Cu1—N1	1.924 (4)	C2—C3	1.380 (5)
Cu1—N1^iv^	1.924 (4)	C3—C4	1.375 (5)
Cu1—Br1	2.654 (12)	C3—H3A	0.9300
Cu1'—N1	1.97 (3)	C4—N1	1.342 (5)
Cu1'—N1^iv^	2.00 (3)	C4—H4A	0.9300
Cu1'—Br1	2.645 (12)	C5—C6	1.377 (5)
Cu2—N2	2.020 (4)	C5—H5A	0.9300
Cu2—Br2	2.5191 (7)	C6—N1	1.342 (5)
Cu2—Br2^v^	2.5191 (7)	C6—H6A	0.9300
Cu2—Br1	2.6275 (9)	C7—N2	1.341 (4)
Cu2—Cu2^v^	2.6889 (17)	C7—C8	1.385 (6)
Br1—Cu2^vi^	2.6276 (9)	C7—H7A	0.9300
Br1—Cu1'^vi^	2.645 (12)	C8—C9	1.391 (4)
Br1—Cu1'^vii^	2.645 (12)	C8—H8A	0.9300
Br1—Cu1'^iv^	2.645 (12)	C9—C8^vii^	1.391 (4)
Br1—Cu1^vi^	2.654 (12)	C9—C10	1.512 (7)
Br2—Cu2^v^	2.5191 (7)	C10—O3^vii^	1.243 (3)
O1—C1	1.260 (4)	N1—Cu1'^iv^	2.00 (3)
O1—Tm1^viii^	2.208 (2)	N2—C7^vii^	1.341 (4)
O2—C1	1.230 (5)		
			
O4—Tm1—O1^i^	109.97 (10)	Cu1'^vi^—Br1—Cu1'^iv^	161.3 (7)
O4—Tm1—O1^ii^	109.97 (10)	Cu1'^vii^—Br1—Cu1'^iv^	180.000 (4)
O1^i^—Tm1—O1^ii^	113.85 (14)	Cu2^vi^—Br1—Cu1^vi^	90.0
O4—Tm1—O5	159.04 (17)	Cu2—Br1—Cu1^vi^	90.000 (2)
O1^i^—Tm1—O5	80.41 (9)	Cu1'—Br1—Cu1^vi^	170.7 (3)
O1^ii^—Tm1—O5	80.41 (9)	Cu1'^iv^—Br1—Cu1^vi^	170.7 (3)
O4—Tm1—O3^iii^	83.02 (12)	Cu2^vi^—Br1—Cu1	90.000 (2)
O1^i^—Tm1—O3^iii^	154.05 (11)	Cu2—Br1—Cu1	90.0
O1^ii^—Tm1—O3^iii^	80.34 (10)	Cu1'^vi^—Br1—Cu1	170.7 (3)
O5—Tm1—O3^iii^	80.85 (11)	Cu1'^vii^—Br1—Cu1	170.7 (3)
O4—Tm1—O3	83.02 (12)	Cu1^vi^—Br1—Cu1	180.000 (1)
O1^i^—Tm1—O3	80.34 (10)	Cu2—Br2—Cu2^v^	64.51 (4)
O1^ii^—Tm1—O3	154.05 (11)	C1—O1—Tm1^viii^	154.2 (3)
O5—Tm1—O3	80.85 (11)	C10—O3—Tm1	139.8 (3)
O3^iii^—Tm1—O3	79.09 (15)	Tm1—O4—Tm1^ix^	135.8 (3)
O4—Tm1—O6	72.25 (17)	Tm1—O4—H4	110.9
O1^i^—Tm1—O6	72.46 (9)	Tm1^ix^—O4—H4	110.9
O1^ii^—Tm1—O6	72.46 (9)	Tm1—O5—H5	120.2
O5—Tm1—O6	128.71 (15)	Tm1—O6—H6B	60.9
O3^iii^—Tm1—O6	133.49 (10)	Tm1—O6—H6C	150.7
O3—Tm1—O6	133.49 (10)	H6B—O6—H6C	105.4
O4—Tm1—H6B	92.1	O2—C1—O1	126.2 (3)
O1^i^—Tm1—H6B	64.0	O2—C1—C2	117.9 (3)
O1^ii^—Tm1—H6B	64.0	O1—C1—C2	115.9 (3)
O5—Tm1—H6B	108.8	C5—C2—C3	118.0 (3)
O3^iii^—Tm1—H6B	140.0	C5—C2—C1	120.8 (3)
O3—Tm1—H6B	140.0	C3—C2—C1	121.2 (3)
O6—Tm1—H6B	19.9	C4—C3—C2	119.5 (4)
Cu1'^iv^—Cu1—N1	93 (4)	C4—C3—H3A	120.2
Cu1'^iv^—Cu1—N1^iv^	89 (4)	C2—C3—H3A	120.2
N1—Cu1—N1^iv^	154.2 (7)	N1—C4—C3	122.6 (4)
Cu1'^iv^—Cu1—Br1	84 (3)	N1—C4—H4A	118.7
N1—Cu1—Br1	102.9 (4)	C3—C4—H4A	118.7
N1^iv^—Cu1—Br1	102.9 (4)	C6—C5—C2	119.7 (3)
Cu1'^iv^—Cu1'—N1	79 (4)	C6—C5—H5A	120.2
Cu1'^iv^—Cu1'—N1^iv^	76 (4)	C2—C5—H5A	120.2
N1—Cu1'—N1^iv^	142.3 (6)	N1—C6—C5	122.4 (4)
Cu1'^iv^—Cu1'—Br1	80.7 (3)	N1—C6—H6A	118.8
N1—Cu1'—Br1	102.0 (9)	C5—C6—H6A	118.8
N1^iv^—Cu1'—Br1	101.2 (9)	N2—C7—C8	123.3 (4)
N2—Cu2—Br2	108.50 (6)	N2—C7—H7A	118.3
N2—Cu2—Br2^v^	108.50 (7)	C8—C7—H7A	118.3
Br2—Cu2—Br2^v^	115.49 (4)	C7—C8—C9	118.9 (4)
N2—Cu2—Br1	122.79 (14)	C7—C8—H8A	120.6
Br2—Cu2—Br1	100.894 (19)	C9—C8—H8A	120.6
Br2^v^—Cu2—Br1	100.894 (19)	C8—C9—C8^vii^	118.1 (5)
N2—Cu2—Cu2^v^	126.47 (15)	C8—C9—C10	120.8 (2)
Br2—Cu2—Cu2^v^	57.744 (18)	C8^vii^—C9—C10	120.8 (2)
Br2^v^—Cu2—Cu2^v^	57.744 (18)	O3—C10—O3^vii^	126.7 (5)
Br1—Cu2—Cu2^v^	110.74 (4)	O3—C10—C9	116.6 (2)
Cu2^vi^—Br1—Cu2	180.0	O3^vii^—C10—C9	116.6 (2)
Cu2^vi^—Br1—Cu1'^vi^	98.6 (4)	C4—N1—C6	117.7 (3)
Cu2—Br1—Cu1'^vi^	81.4 (4)	C4—N1—Cu1	122.3 (4)
Cu2^vi^—Br1—Cu1'^vii^	81.4 (4)	C6—N1—Cu1	119.9 (4)
Cu2—Br1—Cu1'^vii^	98.6 (4)	C4—N1—Cu1'	123.7 (5)
Cu2^vi^—Br1—Cu1'	81.4 (4)	C6—N1—Cu1'	116.5 (5)
Cu2—Br1—Cu1'	98.6 (4)	C4—N1—Cu1'^iv^	117.0 (5)
Cu1'^vi^—Br1—Cu1'	180.0 (13)	C6—N1—Cu1'^iv^	124.3 (5)
Cu1'^vii^—Br1—Cu1'	161.3 (7)	C7—N2—C7^vii^	117.2 (4)
Cu2^vi^—Br1—Cu1'^iv^	98.6 (4)	C7—N2—Cu2	120.8 (2)
Cu2—Br1—Cu1'^iv^	81.4 (4)	C7^vii^—N2—Cu2	120.8 (2)

Symmetry codes: (i) *x*, −*y*+1, −*z*+1; (ii) *x*, −*y*+1, *z*−1/2; (iii) *x*, *y*, −*z*+1/2; (iv) *x*, −*y*+2, −*z*+1; (v) −*x*, −*y*+1, −*z*+1; (vi) −*x*, −*y*+2, −*z*+1; (vii) −*x*, *y*, *z*; (viii) *x*, −*y*+1, *z*+1/2; (ix) −*x*, *y*, −*z*+1/2.

### Hydrogen-bond geometry (Å, °)

**Table:**

*D*—H···*A*	*D*—H	H···*A*	*D*···*A*	*D*—H···*A*
O5—H5···O2^x^	0.81	1.86	2.667 (3)	175.

Symmetry codes: (x) −*x*+1/2, −*y*+3/2, −*z*+1.
